# IFNL4, ACE1, PKR, IFNG, MBL2 genetic polymorphisms and severe COVID-19: A protocol for systematic review and meta-analysis

**DOI:** 10.1097/MD.0000000000029405

**Published:** 2022-05-27

**Authors:** Hengjia Tu, Junrong Bao

**Affiliations:** a Guangzhou Medical University, Xinzao, Panyu District, Guangzhou City, Guangdong Province, People's Republic of China; b Faculty of Big Data and Computing, Guangdong Baiyun University, No.1 Xueyuan Road Jianggao Town, Baiyun District, Guangzhou City, Guangdong Province, People's Republic of China.

**Keywords:** ACE1, corona virus disease 2019, IFNL4, IFNG, genetic polymorphisms, MBL2, meta-analysis, PKR

## Abstract

**Background:**

Corona virus disease 2019 (COVID-19) is caused by SARS-CoV-2, the pathogenic process of SARS-Cov-2 is related to the angiotensin-2 converting enzyme (ACE-2) on host cells. The genetic polymorphisms among different populations may influence the progression of COVID-19. However, the effects of IFNL4, ACE1, PKR, IFNG, and MBL2 in severe COVID-19 have not been systematically assessed.

**Methods:**

We will include all relevant English and Chinese studies by searching the following electronic databases: PubMed, MEDLINE, Embase, Web of Science, Scopus, the Cochrane Library, and Google Scholar before March 31, 2022. Two researchers will independently screen and extract the literature. The methodological quality of the included studies will be evaluated by the Cochrane Handbook for Systematic Reviews of Interventions.

**Result:**

This systematic review and meta-analysis will summarize the association of IFNL4, ACE1, PKR, IFNG, MBL2 genetic polymorphisms, and severe COVID-19. The results will be submitted to a peer-reviewed journal once completed.

**Conclusion:**

The conclusion of our study will provide evidence for the early prevention of severe COVID-19.

**PROSPERO registration number:**

CRD42022301735.

## 1 Introduction

In December 2019, a disease caused by severe acute respiratory syndrome coronavirus type 2 (SARS-CoV-2) was first identified in China and rapidly developed into a global pandemic. SARS-CoV-2, which belongs to the coronavirus family and coronavirus subfamily,^[1]^ has been associated with several clinical conditions involving the lung,^[1]^ intestine,^[2]^ liver,^[3]^ and neurological systems.^[4]^ Some studies^[5,6]^ have shown that the elderly and those with comorbid conditions such as obesity, cardiovascular disease, and diabetes are at higher risk of hospitalization or death from COVID-19, and this association may be related to the pathogenic process of SARS-CoV-2.

The pathogenic process of SARS-Cov-2 is related to the mechanism by which the virus enters the host cell through binding to angiotensin-2 converting enzyme (ACE-2).^[7]^ Currently, it has been proposed that genetic polymorphisms among different populations may influence the progression of COVID-19 reassortment. These genes include HLA, ACE1, OAS-1, MxA, PKR, MBL, E-CR1, FcγRIIA, MBL2.^[8]^ The exact mechanism is not yet clear. These genetic polymorphisms and their differential expression in different populations may lead to differential expression of immune-related proteins, influence the biological process of virus entry into human cells, the process of virus multiplication and the immune response, thus forming a complex interconnection and increasing the tendency of severe COVID-19.^[8,9]^

To the best of our knowledge, there is a lack of systematic evaluation of genetic characterization of severe COVID-19. This study aims to perform a systematic evaluation and meta-analysis of the effects of IFNL4, ACE1, PKR, IFNG, and MBL2 in the course of severe COVID-19. It is expected that genetic profiling can provide ideas for the early identification of people prone to progress to severe COVID-19, which in turn can help in the prevention of severe COVID-19.

## 2 Method

### 2.1 Protocol and registration

This protocol is registered with the International Prospective Register of Systematic Reviews (PROSPERO) under the CRD number CRD42022301735. The proposed systematic review and meta-analysis conforms to the Preferred Reporting Items for Systematic Reviews and Meta-Analyses (PRISMA-P) guidelines.^[10]^

### 2.2 Inclusion criteria

#### 2.2.1 Types of studies

We will include observational studies, case-control retrospective or prospective studies and cross-sectional studies.

#### 2.2.2 Participants

Individuals diagnosed with COVID-19 who are positive for polymerase chain reaction.

#### 2.2.3 Exposure

Differential gene expression and genetics polymorphisms of IFNL4, ACE1, PKR, IFNG and MBL2 in COVID-19 patients.

#### 2.2.4 Outcome

Effect of differential expression of IFNL4, ACE1, PKR, IFNG, and MBL2 on the severity of COVID-19 patients. The main outcomes include hospitalization rates, ICU admissions, and various adverse outcomes including death, respiratory failure, use of assisted breathing measures (tracheal intubation, ventilator, extracorporeal membrane pulmonary oxygen).

### 2.3 Exclusion criteria

Reviews and meta-analyses, letters to the editor, case reports, case series, and expert opinions will be excluded.

### 2.4 Search strategy

We will include all relevant English and Chinese studies by searching the following electronic databases: PubMed, MEDLINE, Embase, Web of Science, Scopus, the Cochrane Library, and Google Scholar before March 31, 2022.

### 2.5 Data collection and analysis

#### 2.5.1 Study selection

**Figure 1:**
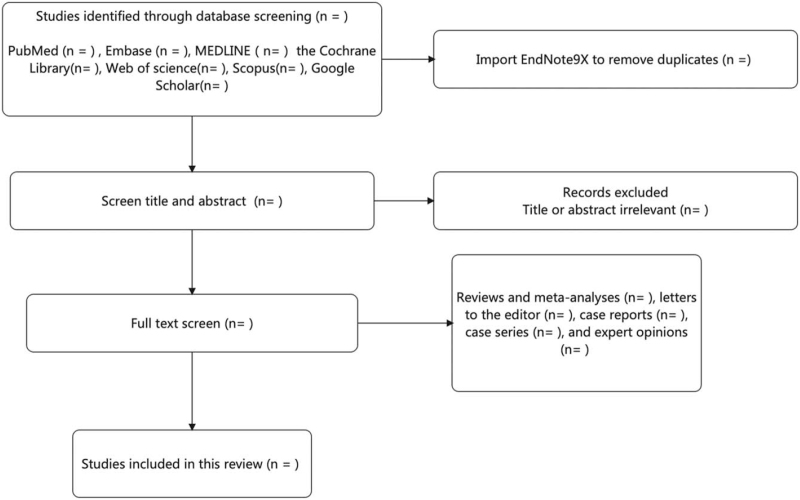
The Preferred Reporting Items for Systematic Reviews and Meta-Analyses flow diagram.

Two authors, HT and JB will independently select the articles, using titles and abstracts. Duplicate studies will be excluded. The same authors will review the text to determine if the studies meet the inclusion criteria. Disagreements will be resolved through discussion. The selection of studies will be summarized in a Preferred Reporting Items for Systematic Reviews and Meta-Analyses flow diagram (Fig. 1).

#### 2.5.2 Data extraction and management

A standardized data extraction form will be developed and tested. Data from each included study will be extracted independently by the reviewers, and any subsequent discrepancies will be resolved through discussion. The data extracted will include information on authors, year of publication, study location (country and continent), type of study, main objectives, population, hospitalization rates, ICU admissions, and various adverse outcomes including death, respiratory failure, use of assisted breathing measures (tracheal intubation, ventilators, epicutaneous membrane lung oxygenation).

Furthermore, participant characteristics (e.g., mean age, gender), and results for the prevalence will be collected.

The study authors will be contacted in case of missing data and/or to resolve any uncertainties. In addition, any additional information will be recorded. All data entries will be checked twice. If we find a set of articles with similar characteristics based on the information in the data extraction table, we will perform a meta-analysis using a random-effects model.

#### 2.5.3 Dealing with missing data

If complete literature or relevant data is not available, we will contact the corresponding author. However, if the missing data cannot be obtained, then the study will be excluded from the analysis.

#### 2.5.4 Assessment of risk of bias

The Cochrane Handbook for Systematic Reviews^[11]^ will be performed to assess a broad category of biases in the included studies. We will evaluate biases from the following 7 aspects: random sequence generation, allocation concealment, blinding of the participants and personnel, blinding of the outcome assessments, incomplete outcome data, selective reporting, and other sources of bias. These studies will be assigned as low risk, high risk, or unclear risk. Inconsistencies will be resolved by discussion with other reviewers.

#### 2.5.5 Assessment of reporting bias

If there are over 10 studies included in the meta-analysis, funnel plots will be used to detect the reporting biases

#### 2.5.6 Assessment of heterogeneity

We will assess the heterogeneity through clinical heterogeneity by the *χ*^2^ test with a *P*-value: if the *P*-value is <.10, then it is statistically significant. We will calculate the *I*^2^ statistic as instructed in the Cochrane Handbook for Systematic Reviews of Interventions. If the *I*^2^ is <40%, then there is small statistical heterogeneity; if it is >75%, there is significantly heterogeneous in the trials, which indicates that they could not be combined, and subgroup analysis is needed.

#### 2.5.7 Data synthesis

We will take advantage of Review Manager software V.5.3 for data analysis and synthesis. Data will be processed with a fixed-effect model if no statistical heterogeneity was observed among the results (*P* ≥ .05 and *I*^2^ ≥ 50%). Meanwhile, the random-effect model will be put into use, if *P* < .05 and *I*^2^ < 50%.

#### 2.5.8 Subgroup analysis

Based on the results of data synthesis, a subgroup analysis or meta-regression analysis will be performed to analyze the source of any heterogeneity.

#### 2.5.9 Quality of evidence evaluation

The quality of evidence will be independently assessed by 2 reviewers and graded for recommendation evaluation, development, and evaluation. Evidence quality will be rated as “high,” “medium,” “low,” or “very low” according to rating criteria based on 5 parameters (publication bias, inconsistencies, inaccuracies, and research limitations).

### 2.6 Ethics and dissemination

Since this study does not involve patient privacy, ethical approval is not required.

## 3 Discussion

To our knowledge, angiotensin-converting enzyme 2 (ACE2) is the cellular receptor^[7]^ for COVID-19 and its differential expression in different individuals influences the replication process of the COVID-19 virus in vivo. COVID-19 first appeared in East Asia but spread rapidly in Europe and caused high mortality.^[12]^ Studies suggest that this feature may be related to genetic polymorphisms of ACE1 and ACE2 in different ethnic groups.^[12–15]^ This feature is specifically manifested by the insertion/deletion polymorphism^[16]^ in the ACE2 gene fragment. This signature may be useful for the assessment of prognostic features^[15]^ of COVID-19 and the therapeutic targeting of potential drugs.^[7,17]^ PKR plays a role in the course of COVID-19 infection, including a regulatory role^[6,18]^ in the virus-induced stress response and induces possible insulin resistance,^[6]^ contributing to the progression of pathophysiological processes in COVID-19 patients with co-morbid diabetes. MBL2 gene polymorphisms^[19]^ are thought to be associated with the severity of COVID-19 infection, and the presence of the B allele (rs1800450)^[20]^ due to MBL deficiency is associated with poorer clinical presentation of patients, including the need for hospitalization and the need for ventilator use.^[20]^ Certain genetic polymorphisms^[21–23]^ in INFL4 may contribute to population susceptibility to COVID-19, but its association with disease severity still requires additional animal studies or clinical studies to reveal. Network pharmacology studies^[24,25]^ have identified multiple pharmacological targets, including INFG, as potential targets for the anti-COVID-19 effects of niacin and have found that patients with colorectal cancer (CRC) may be susceptible^[24]^ to the COVID-19 virus. The study found that vitamin D treatment shortened the length of stay and reduced mortality^[26]^ in COVID-19 cases and INFG played an important role in this process. This study will systematically evaluate the impact of IFNL4, ACE1, PKR, IFNG, and MBL2 in the course of severe COVID-19 using electronic databases and paper-based literature with an eye to the current genetic profile of the severe COVID-19 population. We hope that this study will provide ideas for early identification, prevention, and treatment of severe COVID-19.

## Author contributions

**Conceptualization:** Hengjia Tu, Junrong Bao.

**Data curation:** Hengjia Tu, Junrong Bao.

**Formal analysis:** Hengjia Tu, Junrong Bao.

**Funding acquisition:** Hengjia Tu, Junrong Bao.

**Investigation:** Hengjia Tu, Junrong Bao.

**Methodology:** Hengjia Tu, Junrong Bao.

**Project administration:** Hengjia Tu, Junrong Bao.

**Visualization:** Hengjia Tu, Junrong Bao.

**Writing – original draft:** Hengjia Tu, Junrong Bao.

**Writing – review & editing:** Hengjia Tu, Junrong Bao.
